# Negative-Pressure Wound Therapy Versus Standard Treatment of Adult Patients With Conflict-Related Extremity Wounds: Protocol for a Randomized Controlled Trial

**DOI:** 10.2196/12334

**Published:** 2018-11-26

**Authors:** Andreas Älgå, Sidney Wong, Rawand Haweizy, Kalle Conneryd Lundgren, Johan von Schreeb, Jonas Malmstedt

**Affiliations:** 1 Department of Clinical Science and Education, Södersjukhuset Karolinska Institutet Stockholm Sweden; 2 Department of Public Health Sciences Karolinska Institutet Stockholm Sweden; 3 Operational Centre Amsterdam Médecins Sans Frontières Amsterdam Netherlands; 4 Emergency Management Center Erbil Iraq; 5 The Center for Molecular Medicine Karolinska Institutet Stockholm Sweden

**Keywords:** war-related injuries, negative-pressure wound therapy, extremity wounds, resource-limited settings

## Abstract

**Background:**

In armed conflict, injuries commonly affect the extremities and contamination with foreign material often increases the risk of infection. The use of negative-pressure wound therapy has been described in the treatment of acute conflict-related wounds, but reports are retrospective and with limited follow-up.

**Objective:**

The objective of this study is to investigate the effectiveness and safety of negative-pressure wound therapy use in the treatment of patients with conflict-related extremity wounds.

**Methods:**

This is a multisite, superiority, pragmatic randomized controlled trial. We are considering for inclusion patients 18 years of age and older who are presenting with a conflict-related extremity wound within 72 hours after injury. Patients are block randomly assigned to either negative-pressure wound therapy or standard treatment in a 1:1 ratio. The primary end point is wound closure by day 5. Secondary end points include length of stay, wound infection, sepsis, wound complications, death, and health-related quality of life. We will explore economic outcomes, including direct health care costs and cost effectiveness, in a substudy. Data are collected at baseline and at each dressing change, and participants are followed for up to 3 months. We will base the primary statistical analysis on intention-to-treat.

**Results:**

The trial is ongoing. Patient enrollment started in June 2015. We expect to publish findings from the trial by the end of 2019.

**Conclusions:**

To the best of our knowledge, there has been no randomized trial of negative-pressure wound therapy in this context. We expect that our findings will increase the knowledge to establish best-treatment strategies.

**Trial Registration:**

ClinicalTrials.gov NCT02444598; http://clinicaltrials.gov/ct2/show/NCT02444598 (Archived by WebCite at http://www.webcitation.org/72hjI2XNX)

**International Registered Report Identifier (IRRID):**

DERR1-10.2196/12334

## Introduction

### Background

During armed conflict, injuries commonly affect the extremities, among both civilians [1] and combatants [2]. These injuries are often contaminated with foreign material, increasing the risk of infection [3,4]. Traditionally, conflict-related wounds are surgically treated with debridement of devitalized or contaminated tissue and then covered with a nonadhesive dressing. After 3 to 5 days, the wound is generally examined a second time in the operating room [5].

Negative-pressure wound therapy (NPWT) is widely used in the treatment of wounds and is claimed to promote wound healing and prevent infectious complications. The technique involves covering the wound with a solid foam followed by a plastic film through which a negative pressure is applied. Any wound and tissue fluid is drawn away from the area and collected into a canister. NPWT is suggested by expert consensus for use in a range of surgical applications, including after or in between debridements, as a bridge to definite closure of soft tissue wounds [6]. The technique has previously been used in the treatment of acute conflict-related wounds, but existing reports are retrospective and with limited follow-up [7-10]. Two independent Cochrane reviews of NPWT for the treatment of surgical wounds [11] and traumatic wounds [12] were inconclusive due to the lack of suitably powered, high-quality trials.

### Summary of Potential Risks and Benefits

Both treatment methods (NPWT and conventional dressings) are well established and used in the treatment of acute and chronic wounds. As it is unknown whether there is any difference in outcome between the two treatment modalities, neither patient group may be regarded as receiving preferential treatment. In this study, we will allocate treatment at the end of the first surgery so there is no difference between the groups in terms of surgical risks.

NPWT has not regularly been used at the study sites prior to this study. The introduction of NPWT is not associated with any serious risks compared with standard treatment. Potential benefits of NPWT are shortened healing time and fewer infectious complications. The occlusive dressing may, on the other hand, cause more infections or delay the identification of infection. Other potential risks include pain, mainly associated with dressing changes [13], and bleeding, predominantly minor bleeding from granulation tissue [14]. Conventional wound dressing has the potential benefit of being a safe treatment method used for many years. Since this method permits air into the wound, the risk of health care–associated infections is potentially higher.

### Objective

The objective of this study is to evaluate the effectiveness and safety of NPWT in the treatment of traumatic extremity wounds in a context associated with a high level of contamination and infection.

## Methods

### Study Design

This is a multisite, superiority, pragmatic randomized controlled trial comparing NPWT versus conventional dressing methods in the treatment of patients with conflict-related extremity wounds (ClinicalTrials.gov NCT02444598).

### End Points

The primary end point is wound closure by day 5, by suture, flap, or split-thickness skin graft.

The coprimary end point is net clinical benefit, defined as a composite of wound closure by day 5 and freedom from any bleeding, infection, sepsis, or loss of index limb.

The secondary end points are (1) rate of wound healing, defined as days to wound closure by suture, flap, or split-thickness skin graft; (2) wound infection, defined as purulent discharge [15]; (3) wound size ratio at day 14 (wound size day 14 compared with size day 0, ie, wound healing rate after 14 days); (4) time until wound is deemed no longer requiring professional care; (5) number of surgeries; (6) time to hospital discharge; (7) quality-of-life aspects; (8) wound healing at follow-up days 14 and 30, and at 3 months; (9) bleeding leading to blood transfusion; (10) sepsis; (11) limb amputation (limb with wound included in the study); (12) death; (13) direct health care costs (substudy); and (14) cost effectiveness (substudy).

### Participants

Patients 18 years of age and older presenting at the hospital within 72 hours of sustaining a conflict-related extremity wound are included as they present at the emergency department. In case of multiple wounds, we are selecting the extremity wound with the estimated largest area. Patients are included if they are transferred from another hospital within 72 hours of initial trauma. Patients who present with wounds considered ready for primary closure by suture, flap, or split-thickness skin graft are excluded. Local or systemic infections are treated according to local standard protocols. Wounds in need of debridement are debrided according to International Committee of the Red Cross war surgery protocols [5].

### Setting

Jordan is an upper-middle-income country [16], currently hosting 655,000 Syrian refugees [17]. Médecins Sans Frontières/Doctors Without Borders (MSF), an international nongovernmental organization, runs an emergency trauma project at the Jordan Ministry of Health hospital in Ar Ramtha, 5 km from the Syrian border. Patients within the project receive treatment for blast and gunshot wounds sustained in the Syrian armed conflict. Discharged patients are sometimes continuously treated by MSF in Zaatari refugee camp. The wound infection rate among patients receiving acute surgical treatment at the project has been found to be 11%, with 3 out of 4 patients infected by multidrug-resistant bacteria [18]. Physicians within the project have found wound management to be a major challenge [19]. Patient enrollment in Ar Ramtha, Jordan started in June 2015.

Iraq is an upper-middle-income country [16]. Emergency Hospital is a trauma center in Erbil, Iraqi Kurdistan that is run by a local nongovernmental organization called Emergency Management Center. Most patients receive treatment for conflict-related injuries, and the hospital was one of the key medical institutions receiving the injured from Mosul during the Iraqi offensive against the so-called Islamic State of Iraq and Syria during October 2016 to July 2017 [20]. Patient enrollment in Erbil is ongoing since May 2017.

### Randomization and Blinding

We use a computer-generated randomization code with random variation of 2 fixed block sizes to achieve balance in the allocation of participants to the 2 treatment arms and reduce the opportunity for bias and confounding. Each site has its own dedicated randomization list respecting the 1:1 ratio. The sealed randomization envelopes are opened by the operating room nurse at the end of the first surgery, but before the wound dressing is applied. By randomizing after the operation, we eliminate the risk of treatment allocation influencing the surgeon’s choice of debridement technique. Wound photographs will be evaluated by 2 independent trained evaluators who are blinded to the treatment allocation. Due to the nature of the treatment methods, blinding of the patients or staff involved in the treatment would not be possible.

### Interventions

Patients in the NPWT group receive treatment using a Conformité Européenne–marked professional device with continuous negative pressure of 125 mm Hg. Patients in the control group are treated with conventional wound therapy according to International Committee of the Red Cross war surgery protocols: a nonadhesive dressing covered with a bandage [5]. The exact details of the dressing technique are left to the discretion of the treating surgeon. Dressing details are recorded. All patients receive prophylactic narrow-spectrum antibiotic agents. Fractures are generally immobilized with external fixation. Dressing change frequencies are determined by the treating physician, generally every 3 to 5 days. Any further wound dressing will follow the allocated treatment. Wounds are treated until wound closure. Estimated median duration of treatment (control group) is 5 days.

### Follow-Up Procedures

Follow up is done at each dressing change, at hospital discharge, at days 14 and 30 and at 3 months following the day of randomization (Table 1). Full wound healing or size of wound at the treatment location is noted. Discharged patients either return to the hospital for follow-up or are contacted by phone. If possible, wounds are photo documented and evaluated as described below.

### Quality of Life

We use the 20-item Self Reporting Questionnaire (SRQ-20) to screen for psychological distress [21]. In addition, wound-specific quality-of-life details are recorded, including noise generated by the NPWT pump, movement impairment, skin irritation, odor, sleep quality, discomfort during dressing changes, and pain. SRQ-20 scores and wound-specific quality-of-life details are recorded at baseline and before hospital discharge.

### Sample Size and Power Calculation

The sample size calculation was based on detection of a difference of 25% between treatment groups in the proportion of patients for the primary outcome. We estimated the expected rate of patients reaching the primary outcome at day 5 to be 75% in the NPWT group and 50% in the control group. Based on a power of 80% and a significance level of 5%, we calculated that we would need a minimum sample size of 116 patients (58 per group) to detect a significant difference in the proportions. To adjust for dropouts, we aim to include 200 patients (100 per group).

### Data Collection

Dedicated research nurses collect and enter data into paper-based case report forms during the study period. For all enrolled patients, contact details including mobile phone number are collected. Wounds are photo documented at day 0, at every dressing change, at day 14, and, if possible, at 1 and 3 months’ follow-up. Photo documentation is done in a standardized way with a single-colored background and an adhesive paper ruler attached to the edge of the wound.

**Table 1:** Timeline of trial activities.

Time point	Activity
Baseline	Patient inclusion (consent within 5 days of randomization), patient details, injury details, wound details, photograph of wound, SRQ-20^a^ scores, and quality-of-life details
End of first surgery	Randomization, allocation of treatment, wound details, treatment details, photograph of wound
Dressing change	Treatment details, wound details, photograph of wound
Hospital discharge	Treatment details, wound details, SRQ-20 scores, and quality-of-life details
Day 14	Treatment details, wound details, photograph of wound
Day 30	Treatment details, wound details, photograph of wound
3 months	Treatment details, wound details, photograph of wound

^a^SRQ-20: 20-item Self Reporting Questionnaire.

### Data Management

We will use the EpiData entry software, version 4.4.3.1 (The EpiData Association) to build a database. All data will remain anonymous throughout the data entry and analysis process. Only the research team will know the participants’ names. Identification codes will be safeguarded at the research facilities for the duration of the study.

### Statistical Analysis

We will perform analyses on an intention-to-treat basis with a 2-sided significance level of .05. The primary end point (wound closure by day 5) will be presented as proportions with a 95% confidence interval for the difference in proportions. We will analyze the coprimary end point with standard survival analysis using proportional hazards for comparison of the treatment arms. For the primary end point, we will also perform a per-protocol analysis, excluding patients who did not receive the planned treatment or did not survive to day 5. We will report baseline characteristics as means and standard deviations or numbers and percentages, as appropriate. Subgroup analyses will include age, injury mechanism, initial injury severity, associated fracture versus no fracture, and initial wound size.

### Ethics and Oversight

This study is performed in accordance with the Declaration of Helsinki and the specifications of the International Conference on Harmonisation of Good Clinical Practice. We will report on the trial in line with the Consolidated Standards of Reporting Trials (CONSORT) statement. Ethical approval was given by the Ethics Review Committee of Jordan Ministry of Health (MOH REC 150037) and the Ethics Review Board of MSF (ID 1520) before study initiation in Jordan. We obtained approval from the Research Ethics Committee, Kurdistan Regional Government (2:10 6/3/2017) before study initiation in Iraq. An external monitor regularly inspect the trial master file, monitoring the processes of consent taking, randomization, registration, provision of information, and provision of treatment.

### Informed Consent

Written and oral information in English and Arabic is given to eligible participants. Participants are informed regarding their right to withdraw from the study and issues concerning confidentiality. No incentives or inducements are provided to any participant. Written informed consent before randomization or delayed consent within 5 days of randomization is collected from each patient who agrees to be included.

The principle of delayed consent is an established principle in trials that include critically ill patients and has been considered acceptable from research participants’ perspectives [16]. Due to the nature of the study setting, patients will be transported from the emergency room to the operating room for emergency surgery, often without full consciousness. The emergency circumstances require prompt action and generally provide insufficient time and opportunity to locate and obtain consent from each patient’s legally authorized representative. Therefore we cannot practically carry out the research without the use of delayed consent. Patients who have acute surgery enter the study under presumed consent. Patients are then informed and written consent for continuation in the trial is collected at the fist appropriate time in the postoperative period.

## Results

The trial is ongoing. Patient enrollment started in June 2015. We expect to obtain the results of this trial in 2019.

## Discussion

We present a study protocol for a randomized controlled trial to assess the effectiveness and safety of NPWT use in the treatment of patients with conflict-related extremity wounds. To the best of our knowledge, there has been no randomized trial of NPWT of wounds in this context. We will disseminate the results through peer-reviewed publications. We expect that the findings will increase the knowledge to establish best-treatment strategies.

## Abbreviations

CONSORT: Consolidated Standards of Reporting Trials
MSF: Médecins Sans Frontières/Doctors Without Borders
NPWT: negative-pressure wound therapy
SRQ-20: 20-item Self Reporting Questionnaire

## References

[1] Brennan RJ, Burkle FM, Burkholder BT, Lillibridge SR (1998). Wartime civilian injuries: epidemiology and intervention strategies. J Trauma.

[2] Schoenfeld AJ, Dunn JC, Bader JO, Belmont PJ (2013). The nature and extent of war injuries sustained by combat specialty personnel killed and wounded in Afghanistan and Iraq, 2003-2011. J Trauma Acute Care Surg.

[3] Covey DC, Lurate RB, Hatton CT (2000). Field hospital treatment of blast wounds of the musculoskeletal system during the Yugoslav civil war. J Orthop Trauma.

[4] Fares Y, El-Zaatari M, Fares J, Bedrosian N, Yared N (2013). Trauma-related infections due to cluster munitions. J Infect Public Health.

[5] Giannou C, Baldan M (2010). War Surgery: Working With Limited Resources in Armed Conflict and Other Situations of Violence. Volume 1.

[6] Krug E, Berg L, Lee C, Hudson D, Birke-Sorensen H, Depoorter M, Dunn R, Jeffery S, Duteille F, Bruhin A, Caravaggi C, Chariker M, Dowsett C, Ferreira F, Martínez JMF, Grudzien G, Ichioka S, Ingemansson R, Malmsjo M, Rome P, Vig S, Runkel N, Martin R, Smith J, International Expert Panel on Negative Pressure Wound Therapy [NPWT-EP] (2011). Evidence-based recommendations for the use of negative pressure wound therapy in traumatic wounds and reconstructive surgery: steps towards an international consensus. Injury.

[7] Machen S (2007). Management of traumatic war wounds using vacuum-assisted closure dressings in an austere environment. US Army Med Dep J.

[8] Peck MA, Clouse WD, Cox MW, Bowser AN, Eliason JL, Jenkins DH, Smith DL, Rasmussen TE (2007). The complete management of extremity vascular injury in a local population: a wartime report from the 332nd Expeditionary Medical Group/Air Force Theater Hospital, Balad Air Base, Iraq. J Vasc Surg.

[9] Leininger BE, Rasmussen TE, Smith DL, Jenkins DH, Coppola C (2006). Experience with wound VAC and delayed primary closure of contaminated soft tissue injuries in Iraq. J Trauma.

[10] Hinck D, Franke A, Gatzka F (2010). Use of vacuum-assisted closure negative pressure wound therapy in combat-related injuries--literature review. Mil Med.

[11] Webster J, Scuffham P, Stankiewicz M, Chaboyer WP (2014). Negative pressure wound therapy for skin grafts and surgical wounds healing by primary intention. Cochrane Database Syst Rev.

[12] Iheozor-Ejiofor Z, Newton K, Dumville JC, Costa ML, Norman G, Bruce J (2018). Negative pressure wound therapy for open traumatic wounds. Cochrane Database Syst Rev.

[13] Krasner DL (2002). Managing wound pain in patients with vacuum-assisted closure devices. Ostomy Wound Manage.

[14] Argenta LC, Morykwas MJ (1997). Vacuum-assisted closure: a new method for wound control and treatment: clinical experience. Ann Plast Surg.

[15] Horan TC, Andrus M, Dudeck MA (2008). CDC/NHSN surveillance definition of health care-associated infection and criteria for specific types of infections in the acute care setting. Am J Infect Control.

[16] (2018). World Bank country and lending groups.

[17] (2018). Syria emergency.

[18] Älgå A, Wong S, Shoaib M, Lundgren K, Giske CG, von Schreeb J, Malmstedt J (2018). Infection with high proportion of multidrug-resistant bacteria in conflict-related injuries is associated with poor outcomes and excess resource consumption: a cohort study of Syrian patients treated in Jordan. BMC Infect Dis.

[19] Älgå A, Karlow HK, Alrawashdeh M, Wong S, Khankeh H, Stålsby LC (2018). “Reality rarely looks like the guidelines”: a qualitative study of the challenges hospital-based physicians encounter in war wound management. Scand J Trauma Resusc Emerg Med.

[20] Nerlander MP, Haweizy RM, Wahab MA, Älgå A, von Schreeb J (2018). Epidemiology of trauma patients from the Mosul Offensive, 2016–2017: results from a dedicated trauma center in Erbil, Iraqi Kurdistan. World J Surg.

[21] Beusenberg M, Orley J (1994). A user's guide to the Self Reporting Questionnaire (SRQ).

